# Sol-gel synthesis of lithium doped mesoporous bioactive glass nanoparticles and tricalcium silicate for restorative dentistry: Comparative investigation of physico-chemical structure, antibacterial susceptibility and biocompatibility

**DOI:** 10.3389/fbioe.2023.1065597

**Published:** 2023-04-03

**Authors:** Hazel O. Simila, Aldo R. Boccaccini

**Affiliations:** Institute of Biomaterials, Department of Materials Science and Engineering, University of Erlangen-Nuremberg, Erlangen, Germany

**Keywords:** tricalcium silicate, bioactive glass nanoparticles, property evaluation, sol-gel, lithium, restorative dental biomaterial

## Abstract

**Introduction:** The sol-gel method for production of mesoporous bioactive glass nanoparticles (MBGNs) has been adapted to synthesize tricalcium silicate (TCS) particles which, when formulated with other additives, form the gold standard for dentine-pulp complex regeneration. Comparison of TCS and MBGNs obtained by sol-gel method is critical considering the results of the first ever clinical trials of sol-gel BAG as pulpotomy materials in children. Moreover, although lithium (Li) based glass ceramics have been long used as dental prostheses materials, doping of Li ion into MBGNs for targeted dental applications is yet to be investigated. The fact that lithium chloride benefits pulp regeneration *in vitro* also makes this a worthwhile undertaking. Therefore, this study aimed to synthesize TCS and MBGNs doped with Li by sol-gel method, and perform comparative characterizations of the obtained particles.

**Methods:** TCS particles and MBGNs containing 0%, 5%, 10% and 20% Li were synthesized and particle morphology and chemical structure determined. Powder concentrations of 15mg/10 mL were incubated in artificial saliva (AS), Hank’s balanced saline solution (HBSS) and simulated body fluid (SBF), at 37°C for 28 days and pH evolution and apatite formation, monitored. Bactericidal effects against *S. aureus* and *E. coli*, as well as possible cytotoxicity against MG63 cells were also evaluated through turbidity measurements.

**Results:** MBGNs were confirmed to be mesoporous spheres ranging in size from 123 nm to 194 nm, while TCS formed irregular nano-structured agglomerates whose size was generally larger and variable. From ICP-OES data, extremely low Li ion incorporation into MBGNs was detected. All particles had an alkalinizing effect on all immersion media, but TCS elevated pH the most. SBF resulted in apatite formation for all particle types as early as 3 days, but TCS appears to be the only particle to form apatite in AS at a similar period. Although all particles had an effect on both bacteria, this was pronounced for undoped MBGNs. Whereas all particles are biocompatible, MBGNs showed better antimicrobial properties while TCS particles were associated with greater bioactivity.

**Conclusion:** Synergizing these effects in dental biomaterials may be a worthwhile undertaking and realistic data on bioactive compounds targeting dental application may be obtained by varying the immersion media.

## 1 Introduction

Biomaterials used in dental treatment can be based on metals, polymers, ceramics or composites (O’Brien, 2002; Sakaguchi et al., 2019). The ability of new materials for restorative dentistry to be bioactive, yet antibacterial and biocompatible, is a key driver of research and development (R&D) of novel material products (Rekow et al., 2013; Huang et al., 2020; Iftikhar et al., 2021). The manner in which individual compounds for potential use in new dental materials are evaluated should consider the performance of already successful materials, but even more importantly the intricate nature of the environment in which they will be used. A recent review on bioceramics in dentistry highlighted the paucity of data on bioactive glass in dentistry, while highlighting the need for understanding the unique oral environment and its impact on bioceramics (Chitra et al., 2022). Therefore, comparative investigations of various bioceramics represents an under researched, yet foundational topical area. Although many bioceramic compositions can be studied, this research focuses on tricalcium silicate (TCS) particles and mesoporous bioactive glass nanoparticles (MBGNs) synthesized through a common sol-gel route.

Tricalcium silicates respond to the chemical formula—Ca_3_SiO_5_ (3CaOSiO_2_) Most literature simplifies and abbreviates this to C3S or TCS. Routine synthesis often yields a mix of both TCS and dicalcium silicate (DCS) whose formula is (Ca_2_SiO_4_) and often, both are simply referred to as calcium silicates (Primus et al., 2019). Since the 1990s, TCS dental cements have enjoyed increasing adoption as materials of choice pulp-dentine regeneration (Torabinejad and White, 1998). Most of the TCS compounds used in these materials were often derived from naturally occurring minerals (Primus et al., 2019), however the end products were often laden with impurities (Lee et al., 2018; Abdalla et al., 2020). It is only recently that purer approaches to produce TCS by proprietary technologies, such as Active Biosilicate Technology™ (Septodont, 2009) have emerged, Additionally, a more controlled laboratory procedure that applies sol-gel nanotechnology is beginning to research more closely, the *in vitro* properties of pure TCS (Lee et al., 2018). Despite the indisputable biocompatibility and bioactivity of TCS, their ability to suppress bacteria growth remains a topic of discussion (Farrugia et al., 2017).

MBGNs on the other hand are a class of bioactive glasses (BAG) synthesized by sol-gel method that feature unique surface characteristics which may explain the ability of MBGNs to inhibit microbial growth and enhance tissue regeneration (Vichery and Nedelec, 2016). Ion incorporation into BAG is not uncommon to achieve specific biological activity (Hoppe et al., 2011). In this context, however, incorporation of Li into MBGNs for target bioactive dental materials is still under researched (Simila and Boccaccini, 2022). This is despite the successful commercialization of several Li based bioceramics used in prosthetic dentistry (Daguano et al., 2019; Dahiya et al., 2019). Moreover previous research has shown lithium chloride (LiCl) to hold potential as a successful agent for dentine pulp regeneration (Ishimoto et al., 2015). Historically, Li was discovered to possess unique antimicrobial and anti-inflammatory properties following unrelated application in psychiatric therapy (Lieb, 2007). Subsequently, it is eliciting significant interest in the field of doped glasses for tissue engineering (Moghanian et al., 2017; Haro Durand et al., 2019) but available research on the same remains scarce. Two related studies include one on melt derived BAG (Khorami et al., 2011) and another on scaffolds (Miguez-Pacheco et al., 2016) for bone regeneration—none of which were produced by the wet chemical, sol-gel process.

The evaluation of new particles that satisfy desirable criteria for inclusion as constituents of bioactive dental biomaterials should attempt to assess the new particles with special consideration of the oral environment (Simila and Boccaccini, 2022). In this particular study, bioactivity is considered according to the definition provided by Larry Hench in 1969 (Hench et al., 1971). Thus, we mainly position our experiments within the context of the ability of the materials being investigated to form apatite when immersed in representative body fluids. It is possible and desirable that these materials may also be able to stimulate a specific biological response that is consistent with osteogenic/odontogenic differentiation. The latter processes have been considered to represent an alternative (Vallittu et al., 2018) and more accurate definition of bioactivity according to (Darvell and Smith, 2022). Generally, the term has evolved over time to encompass the ability of a material to dissolve and release ionic products capable of mineralization while having a positive effect on the surrounding tissues and biological processes (Melo et al., 2021).

Often, SBF is used to evaluate bioactivity since its composition bears similarities to human blood plasma (Kokubo and Takadama, 2006). In the case of particles targeting application in the oral cavity, keeping in mind the tooth and oral microenvironment suggests the necessity to test this property using alternative immersion fluids. In the case of biomaterials in contact with saliva, artificial saliva (AS) becomes the natural alternative. It also becomes important to compare the chemical behavior of the particles in Hank’s balanced saline solution (HBSS) since most research on TCS based dental cements has advocated testing in this fluid (Grech et al., 2013; Kebudi Benezra et al., 2017). Although most of the ions are present across all the fluids, their concentrations differ as captured in Table 1. For example, CaCl_2_ ion concentration is only 0.13 g/L in AS, 0.14 g/L in HBSS, but almost double this amount in SBF. Typically, AS also lacks hydroxycarbonate and sulphate ions.

**TABLE 1:** Overview of the composition of the three immersion media used in the study.

Constituents	Immersion media (concentration = *g*/L)		
	AS**#**	HBSS	SBF
Calcium Chloride (CaCl_2_)	0.13	0.14	0.292
Magnesium Chloride (MgCl_2_.6H_2_O)	0.05	0.1	0.311
Magnesium Sulfate (MgSO_4_-7H_2_O)	-	0.1	-
Potassium Chloride (KCl)	1.2	0.4	0.225
Potassium Phosphate (K_2_HPO_4_.3H_2_0)	0.35	0.06	0.231
Sodium Bicarbonate (NaHCO_3_)	-	0.35	0.355
Sodium Chloride (NaCl)	0.85	137.9	8.035
Sodium Phosphate dibasic (Na2HPO4) anhydrous	-	8	-
D-Glucose (Dextrose)	-	10	-
Sodium sulphate (Na_2_SO_4_)	-	-	0.072
IM hydrochloric acid	-	-	39–44 mL
Tris Buffer	-	-	6.118
Mucin	3.5%	*	-
Methylparaben/Methylhydroxybenzoate*	0.8	-	-
Benzalkonium chloride	0.02	-	-
EDTA	*	-	-
Xylitol	*	-	-
Peppermint and spearmint oils	*	-	-

^*^ Exact values not available **#** Composition derived from Reijden et al. (1996).

Comparison of the *in vitro* behavior of TCS, MBGNs and Li-doped MBGNs in a single study is worth undertaking. MBGNs continue to provide very promising *in vitro* and *in vivo* results but are yet to be successfully incorporated into actual patient care (Simila and Boccaccini, 2022). On the contrary, TCS based materials are the current most recommended products for promoting dentine regeneration in clinical dental practice, with a proven track record of good performance (Guivarc’h et al., 2020). Preliminary application of pulp capping material based on bioactive glass in children affected by pulpitis, led to unexpected negative outcomes in one of the latest clinical trials (Elhamouly et al., 2021). Beyond the reasons portrayed by the authors for the undesirable results of the trial, the conditions of synthesis of BAG must be also considered as they influence the characteristics of the nanoparticles affecting also their biological performance (Simila and Boccaccini, 2022). Therefore, preclinical research should strive towards providing detailed data comparing constituents of already successful dental materials such as those based on TCS particles with more novel particles such as MBGNs. Only alongside such efforts, can MBGNs make a jump forward towards clinical application as has been the case with other bioactive glass compositions in the past (Cannio et al., 2021). Indeed, this would reflect expert opinion concluding that *in vitro* research impacts progression towards successful clinical trials of novel therapies (Hammel et al., 2022).

Therefore, for the first time we present the results of a comparative analysis of the physical, chemical and biological behavior of TCS and 70Si30Ca MBGNs with and without Li ion doping, produced by sol-gel method. Although sol-gel synthesis of TCS based compounds is not entirely new (Zhao and Chang, 2004), we propose that by comparing the behavior of these particles in AS, HBSS and SBF, the obtained results can subsequently be used to interpret the outcomes related to bioactivity, antibacterial and cell viability evaluations. Collectively, this information could help predict performance when the biomaterials are used as adjuvants in select restorative dental biomaterial applications.

Therefore, the aim of this exploratory experimental study was to synthesize TCS and Li doped MBGNs by sol-gel method and conduct parallel characterizations of the obtained particles in terms of morphology, bioactivity, and pH behaviour in three dissolution media. Comparative antimicrobial properties and biocompatibility were also assessed.

## 2 Materials and methods

### 2.1 Synthesis of the TCS and MBGNs

Synthesis precursors and steps are summarized in Supplementary Table S1. TCS was synthesized by sol-gel method (Balbinot et al., 2020). Initially, 200 µL of 65% nitric acid (HNO_3_) (Sigma, Germany) was dissolved in 15 mL of deionized (DI) water (MilliQ) to act as a catalyst. 8.5 mL of tetraethyl orthosilicate (TEOS (Sigma, Germany) was added and stirred for 30 min at room temperature, before sequential addition of 26.5 g of calcium nitrate tetrahydrate (Sigma, Germany). The complete mixture was kept under stirring at 500 rpm, for 1h, at room temperature. The formed sol was transferred to a 60°C oven for 24 h for aging and thereafter, the obtained gel was dried at 120°C for a further 24 h prior to calcination at 700°C for an additional 24 h. Retrieved powder was dry milled in a milling device (Retsch PM 100, Retsch GmbH, Germany). Concurrently, MBGNs of nominal composition (mol%) 70SiO_2_-30CaO, 70SiO_2_-25CaO-5Li, 70SiO_2_-20CaO-10Li and 70SiO_2_-10CaO-20Li were synthesized using a microemulsion based sol-gel method. Typically, 5.6 g of cetrimonium bromide (CTAB) (Merck, Germany) was initially dissolved at 35°C in 264 mL of DI water (MilliQ) under continuous stirring. 10–20 min later, when the solution was clear, the temperature was lowered to 20°C and 80 mL of ethyl acetate (EA)-99% (Sigma, Germany) was added. 30 min later, 56 mL of aqueous ammonia was introduced into the solution (1 M) (VWR, France), followed by 28.8 mL of TEOS, 15 min later. In the final stage, calcium nitrate tetrahydrate (Ca (NO_3_)_2_.4H_2_O) was added slowly, in small increments, to allow dissolution of one batch, before adding the next. This lasted around 30 min before the corresponding lithium nitrate (LiNO_3_) was dissolved to yield the doped MBGNs. All these stages occurred under constant stirring. The final solution was then stirred for four more hours. The resulting suspension was centrifuged for 15 min at 7830 rpm (Centrifuge 5430R, Eppendorf, Germany) to retrieve the nanoparticles which were rinsed twice with DI water and once with 96% ethanol. The final deposits collected were dried overnight in a 60°C oven followed by calcination at 700°C for 3 h (2°C/min). All chemicals were purchased and used as received without further purification. Additional physico-chemical, biological, and antibacterial behavior was evaluated as described below.

### 2.2 Particle characterization

Retrieved particles were subjected to further characterization of morphology and elemental composition. To determine the morphology, scanning electron microscopy (FESEM, Auriga Crossbeam, Carl Zeiss Microscopy, GmbH, Jena, Germany) was employed. Obtained SEM micrographs were imported into an image analysis software (ImageJ, NIH, United States) to estimate the average particle size and distribution (*n* = 30). Li content in the doped MBGNs particles was also assessed by inductively coupled plasma optical emission spectroscopy (ICP-OES). Molecular analysis of the TCS and MBGN powder particles was conducted by Fourier transform infrared spectroscopy (FTIR) (SHIMADZU, IRAffinity-1S spectrophotometer, Shimadzu Corp, Tokyo, Japan). 40 scans at a resolution of 4 cm^-1^ in the 400 to 4000 cm^-1^ wavelength range were captured in absorbance mode. Structural analysis of the powders was done by x-ray diffraction (XRD) (Miniflex 600 HR, Rigaku, Japan) using 40 kV copper K-α radiation. The data were obtained in the 2Ɵ range, between 10 and 70. A step size of 0.020 and speed of 4^o^ per minute were employed. Both FTIR and XRD data were normalized to 0–1, prior to graphing as relative absorbance and intensity, respectively.

### 2.3 In vitro bioactivity in three immersion media

Three different dissolution media (Supplementary Table S1) were used to evaluate the bioactivity trends of the five particles. Whereas AS (pH 5.8: Pharmadan A/S Denmark), and HBSS (pH 7.8: Gibco, Germany) were used as received, SBF (pH 7.4) was prepared following the recommended protocol by Kokubo and Takadama (2006). Individual TCS and MBGN powders (*n* = 3) were weighted and introduced into sterile 25 mL falcon tubes containing 10 mL of the respective dissolution media at 37°C (1.5 mg/mL ratio). These were incubated in an orbital shaker (IKA® KS 4000i, Germany) at 37 ± 1°C and were gently agitated at 90 rpm. At the end of the predetermined immersion periods of 1 day, 3 days, 7 days, 14 days, 21 days and 28 days, the solutions were centrifuged to separate the supernatant from the remaining particles. The later were rinsed with deionized water and 99% ethanol before being dried in an oven at 60°C for 1 day. Dry powders were appropriately labelled based on particle type, immersion media and time point and selected samples characterized by SEM, XRD and FTIR following the description in 2.2 above. Commercial hydroxyapatite nano powder (Sigma, Germany) was analyzed alongside the powders of interest to act as reference for apatite.

### 2.4 pH in AS, HBSS and SBF

Parallel to the bioactivity assessments, pH changes during incubation were also examined. After establishing the initial pH of the media before immersion as follows; AS (pH 5.8), HBSS (pH 8.0) and SBF (pH 7.4), the pH of the same solutions would be measured periodically at 1 day, 3 days, 7 days, 14 days, 21 days and 28 days using a pH meter (Jenway™ 3510, Thermo Fischer).

### 2.5 Antibacterial evaluation

The antibacterial ability of the sol-gel synthesized particles against *Escherichia coli (E.coli)* and *Staphylococcus aureus* (*S. aureus*) was assessed by the broth dilution method. As a first step, 2 g of each particle type was weighed and sterilized at 160°C for 2 h. The sterile powder was then immersed in 20 mL of sterile lysogeny broth (LB, Luria/Miller) medium and incubated for 24 h, at 37°C under constant agitation of 120 rpm. Concurrently, bacterial suspensions were prepared by inoculating approximately 10 mL of LB medium with each strain of bacteria, before incubating at 37°C for 24 h. The ideal optical density (OD) of the bacteria suspensions to correspond with 0.015 (approximately 1 × 107 colony forming units (CFUs)/ml was individually determined by dispensing *x* µl of the suspension into 1 mL of LB medium and measuring the absorbance. Subsequently, 15 µL volume of the bacteria suspension was co-cultured with 2 mL of the previously prepared particle eluates for 48 h. OD measurements were recorded at 3 h, 24 h and 48 h. A reference blank was prepared from medium whereas the bacterial suspension in LB medium acted as the control. Each measurement was repeated thrice. To convert the OD reading to relative bacteria viability, the equation below was used:

$$Relative\;bacteria\;viability\;\left(\%\right)=\frac{OD\;Sample}{OD\;Control}x100$$

### 2.6 In vitro cell viability

Testing for cytocompatibility was performed using MG63, human osteoblast like cells obtained from European Collection of Authenticated Cell Cultures (ECACCS, Sigma Aldrich, Darmstadt, Germany). The cells were maintained in Dulbecco’s Modified Eagle Medium (DMEM) (Gibco®, Thermo Fisher Scientific, United States), supplemented with 10% fetal bovine serum (FBS) and 1% penicillin-streptomycin (PS) antibiotic, in 75cm^2^ cell culture flasks. The cell cultures were preserved in a humidified atmosphere of 95% air and 5% CO_2_ at 37°C throughout and were routinely trypsinized and sub-cultured before the actual cytotoxicity experiments which were conducted in accordance with the indirect method outlined in ISO 10993–5. The glass samples, previously sterilized at 160°C for 2 h, were weighed and placed in pure DMEM at concentrations of 10 mg/mL, before being incubated for 24 h at 37°C. Concurrently, the confluent cells were counted and seeded at a density of 100,000cells/well in 24 multi-well plates for 24 h at 37°C. The extracts were filtered in a sterile environment and powder concentrations of 1 mg/mL^−1^ and 0.1 mg mL^−1^ prepared for evaluation. Cells without treatment with powders were used as a control. The fluid extract was applied to a pre-cultured cell monolayer and jointly incubated for 2 days under CO_2_ atmosphere at 37°C. Positive and negative controls were respectively prepared by seeding cells of the same density, with culture medium or with culture medium containing dimethyl sulfoxide (DMSO). At the end of the incubation period, the medium was removed, and cells were rinsed with phosphate buffered saline (PBS) before measuring mitochondrial activity of the cells in contact with extract fluids using WST-8 method (CCK-8 Kit, Sigma Aldrich, Germany). Thus, 400 uL of WST-8 solution (1% v/v) was added to the wells and incubation continued for 2 h until colour change was observed. 100 μL of the medium was transferred to a separate 96 multi-well plate and optical absorbance measured at *λ* = 450 nm in a multi-mode microplate reader (PHOmo, Anthos Mikrosysteme GmbH, Germany). Since the amount of formazan produced is directly proportional to the number of living cells in culture, relative cell viability was calculated according to the equation below:

$$Mean\;cell\;viability\;\left(\%\right)=\frac{Absorbance\;of\;test\;sample-Absorbance\;of\;blank}{Absorbance\;ofpositive\;control-absorbance\;of\;blank}\;X\;100$$

All experiments were performed in triplicate.

After completing the WST assays, haematoxylin and eosin (H&E) staining methods were employed for analysing the cell density. Briefly, the attached cells in the 24 multi-well plates were washed with PBS before being fixed with a 4% w/v paraformaldehyde solution for 15 min. This was followed by rinsing with DI water and staining with Haematoxylin 300ul/well for 10 min before washing again with tap water and Scott’s tap water for 5 min. The cells were further stained with 0.4% eosin stain (in a saturated aqueous solution of 60% ethanol and 5% acetic acid) for 5 min. Finally, the stained cells were dehydrated with 95% and 99% ethanol and air-dried in a fume hood prior to cell density examination using a light microscope (Primo Vert, Carl Zeiss).

## 3 Statistical analysis

Where applicable, results were presented as mean ± standard deviation of the mean (SD). For multiple comparisons, data were analyzed using Pearson’s Comparison which is a two-way ANOVA or one-way ANOVA and the Tukey *post hoc* test using Origin (OriginLab, Northampton, MA, United States). Significant differences between the groups were assumed if *p* was <0.05.

## 4 Results

### 4.1 Particle synthesis and characterization

**FIGURE 1:**
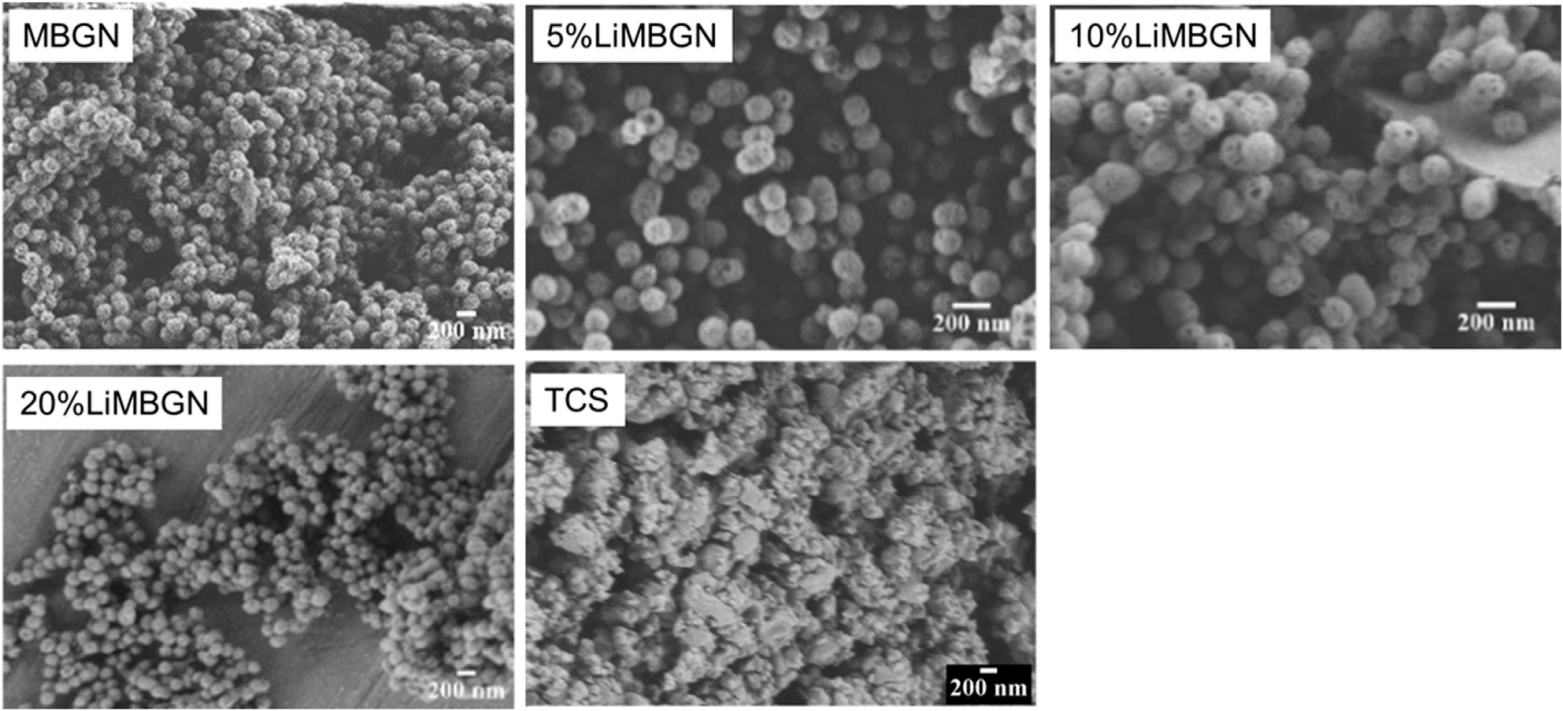
SEM images of freshly synthesized MBGNs and TCS prior to immersion.

**FIGURE 2:**
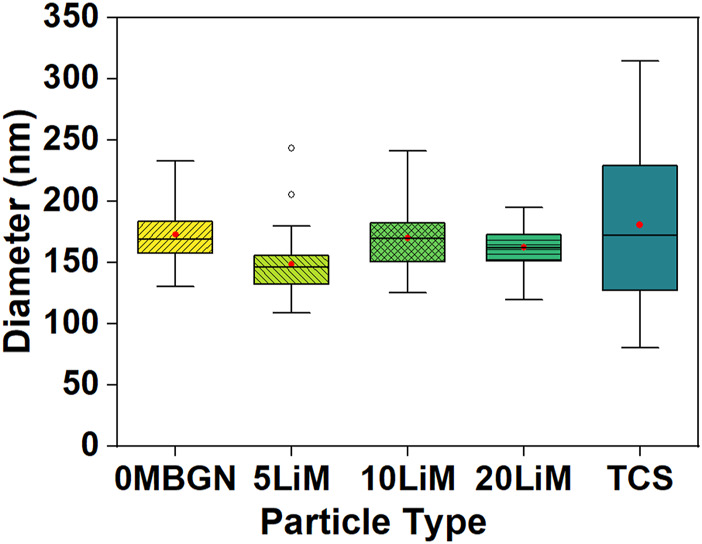
The particle size distribution of the as synthesized particles.

All the powders produced were white in color. In terms of microstructure, all MBGNs were spheres with surface pores whereas TCS formed irregular agglomerates (Figure 1). Particles sizes for MBGNs, 5Li-MBGNs, 10Li-MBGNs and 20Li-MBGNs were 172 ± 21 nm, 148 ± 25 nm, 169 ± 25 nm, 162 ± 17 nm respectively (Figure 2). Although TCS particle size was harder to resolve due to agglomeration, it was found to lie in the 115–240 nm range for individual particles within micron sized agglomerates. MBGNs were smaller and more inform in size compared to TCS particles which were generally larger and with wider size variability.

**FIGURE 3:**
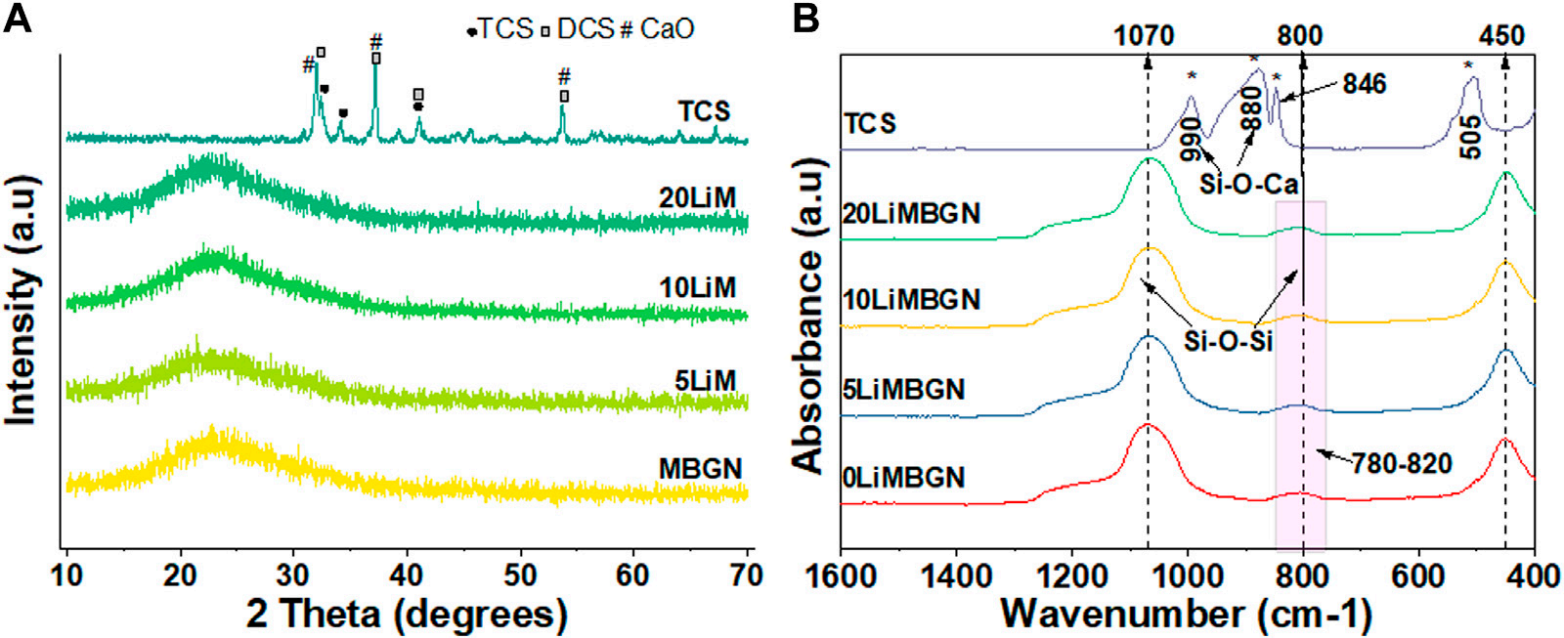
Comparative XRD patterns **(A)** and FTIR spectra **(B)** of as synthesized particles. MBGNs are seen to be amorphous. Peaks marked with asterisks at 880 cm^−1^, 990 cm^−1^, 846 cm^-1^ and 505 cm^-1^ **(B)** are analyzed in the text.

Analysis of the mean particle sizes revealed a statistically significant difference between 5LiMBGNs and TCS only (*p* > 0.01). Overall, Li doping did not seem to significantly impact the morphological characteristics of the MBGNs. In terms of relative composition of the MBGNs, ICP-OES revealed low Li ion incorporation into MBGNs, as shown in Table 2. XRD confirmed the amorphous nature of the MBGNs, while revealing intense peaks associated with overlapping TCS (32.4°, 34.2°, 41.1°) and DCS (32.0°, 37.4°, 41.2°53.5) crystals in TCS. Some calcium oxide (CaO) peaks were also detected at approximately 31.9, 37.2 and 53.6 2θ (Figure 3). From the FTIR spectra of TCS, Si-O-Ca bonds marked with asterisks could be assigned to the split peaks at 880 cm^−1^ and 990 cm^−1^ while O-Si-O stretching vibrations and Si-O-Si bending vibrations are present at around 846 cm^−1^ and in the 505 cm^−1^ region respectively (Lippincott et al., 1958; Lee et al., 2018). Peak lines at around 450 cm^−1^ attributed to Si-O-Si rocking, Si–O–Si stretching vibrations at 1070 cm^−1^ and Si–O–Si bending vibration at 800 cm^−1^ are uniformly present in the glass particles (Aguiar et al., 2009).

**TABLE 2:** Composition by weight percentage of the Li doped MBGNs according to ICP-OES.

Composition (weight)	Doped MBGNs		
	5LiMBGNs	10LiMBGNs	20LiMBGNs
SiO_2_%	88.68	87.86	92.65
CaO %	8.48	6.69	4.67
Li_2_O %	0.03	0.07	0.11

### 4.2 Comparative pH in artificial saliva, HBSS and SBF

**FIGURE 4:**
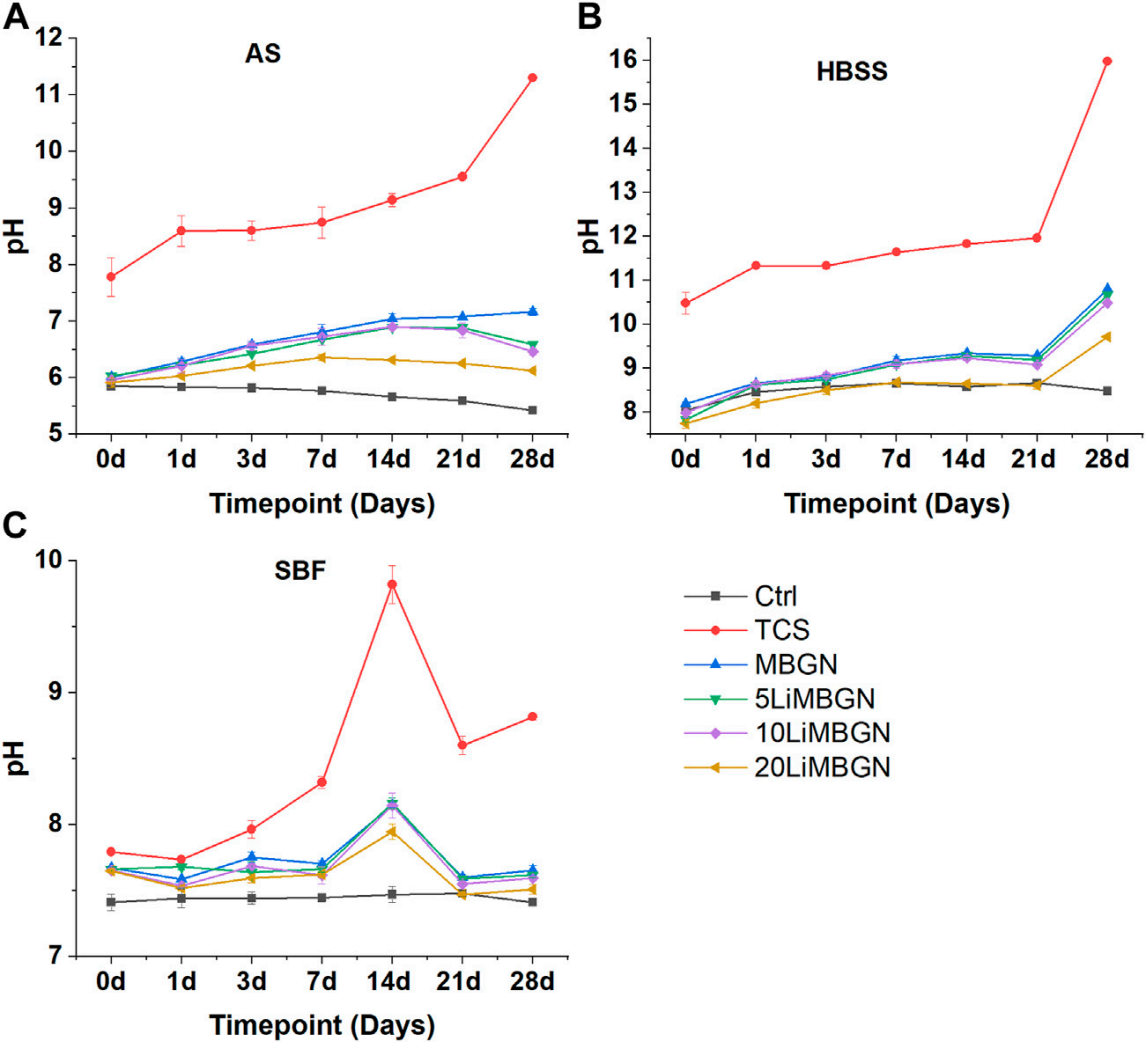
pH evolution of the five particle types, as a function of time following immersion in three media—**(A)** in AS, **(B)** in HBSS and **(C)** in SBF.

All particles had an alkalinizing effect on immersion media as shown by an immediate rise in pH as early as the first hour for TCS following immersion in AS and HBSS and within a day for the MBGNs group immersed in AS and HBSS. The pH for all the particles appears to rise slightly in SBF during the first hour, before dropping slightly on the first day (Figure 4). Following the pH changes recorded in AS and HBSS during the first hour and on 1 day, the subsequent measurements at 3 days, 7 days, 14 days and 21 days were relatively stable, before sharply increasing for TCS at 28 days.

A similar pattern is observed for the MBGNs in HBSS. On the contrary, pH was highest at day 14 in SBF, before dropping again on 21 days and remaining relatively unchanged till 28 days. It was also observed that TCS elevated pH the most, at all time points. The highest value for TCS was observed on day 28 in HBSS, where it stood at 15.98, compared to a beginning pH of 10.48 which was measured within an hour of immersion. Within the mesoporous particles, it appears as though the higher the degree of doping, the lower the pH as seen with 20LiMBGNs recording consistently low pH, while the opposite is true for undoped MBGNs. This variation is obvious in AS, and less so in HBSS and SBF, where any differences in pH between MBGNs, 5LiMBGNs and 10LiMBGNs are almost imperceptible. Overall, pH was highest in HBSS, and lowest in AS. The higher pH values measured for TCS relative to the MBGNs are not as pronounced following immersion in SBF.

### 4.3 SEM, XRD and FTIR characterization of *in vitro* bioactivity in artificial saliva, HBSS and SBF

**FIGURE 5:**
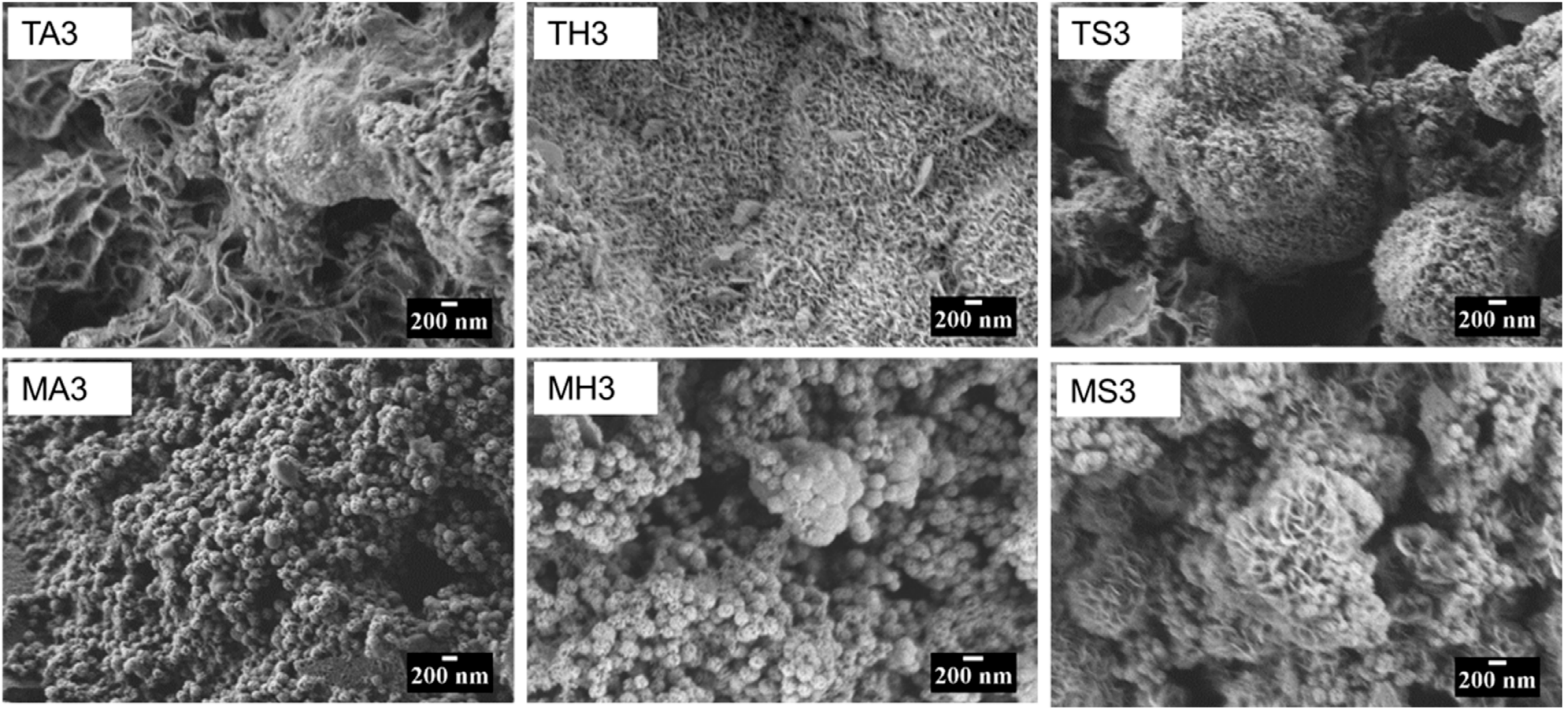
Panel comparing the changes in the particle morphology of TCS (top) and MBGNs Bottom) at day 3 of immersion in AS, HBSS and SBF. The micrographs are arranged from right to left in AS (left), HBSS (middle) and SBF (right).

**FIGURE 6:**
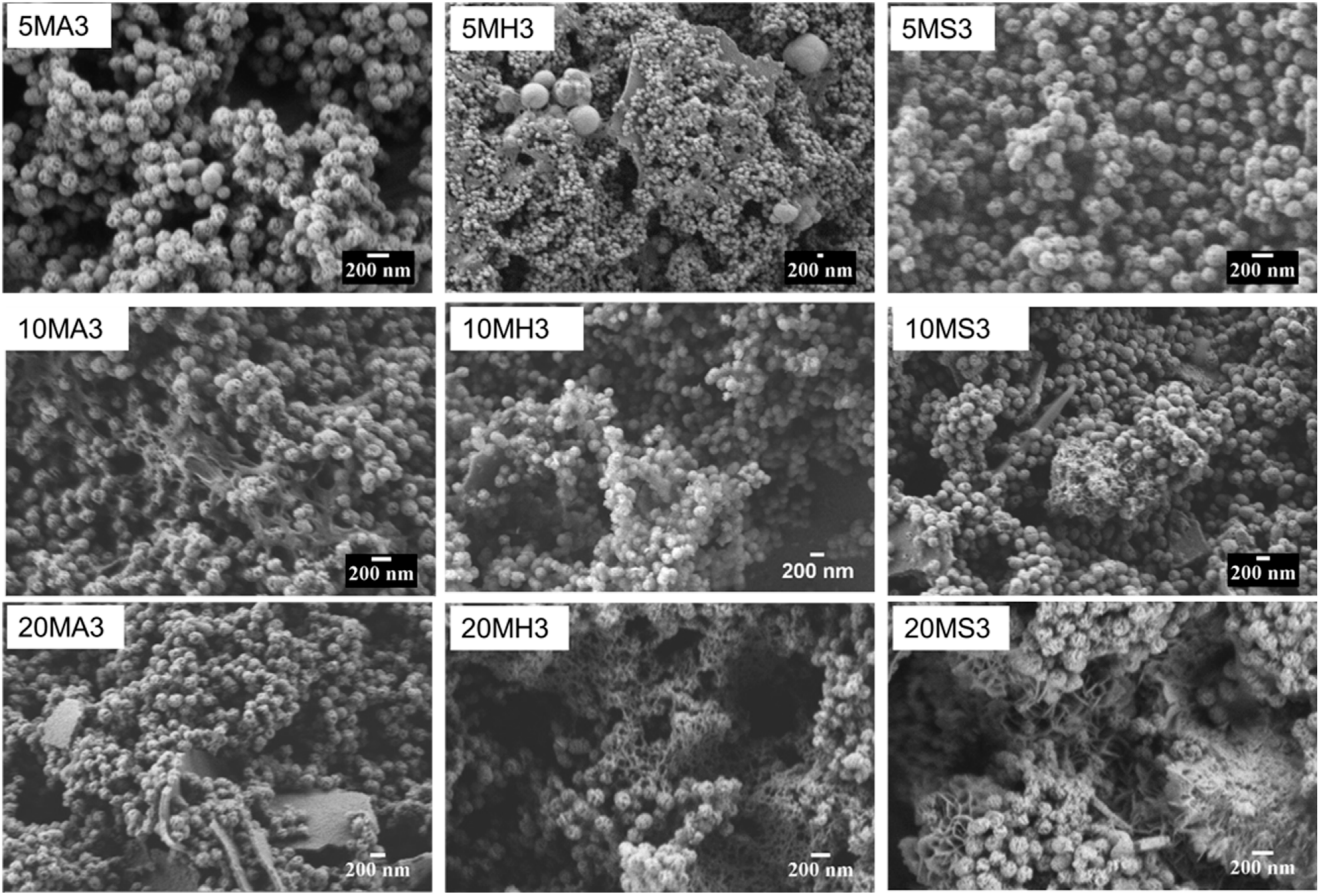
SEM micrographs of the doped MBGNs arranged in rows as 5LiMBGNs (top), 10LiMBGNs (middle) and 20LiMBGNs (bottom) at day 3 of immersion. The micrographs are arranged from right to left based on immersion fluid AS (left), HBSS (middle) and SBF (right).

Changes to the morphology of the particles following immersion were easily compared based on SEM images. Relevant micrographs at day 3 are shown in Figure 5 and Figure 6. In TCS in AS, the dominant morphology is sheet-like, while small cauliflower-like globules, covered by needle-like crystals of what can be assumed to be HA crystals are more visible on the surfaces of all the remaining TCS samples immersed in HBSS and SBF. Such morphology changes are common in MBGNs, whose apatite formation is only identifiable at day 3 in HBSS and SBF. For the doped glasses as well, features of apatite cannot be detected in the AS group but become more obvious in the other fluids. The pattern remains similar in the SEM images for the other time points not shown here.

**FIGURE 7:**
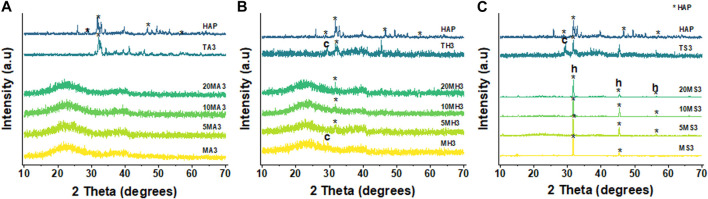
XRD patterns of all the particles following immersion for 3 days in AS **(A)**, HBSS **(B)** and SBF **(C)** where * refers to the HAP, h represents halite, and c refers to calcite.

**FIGURE 8:**
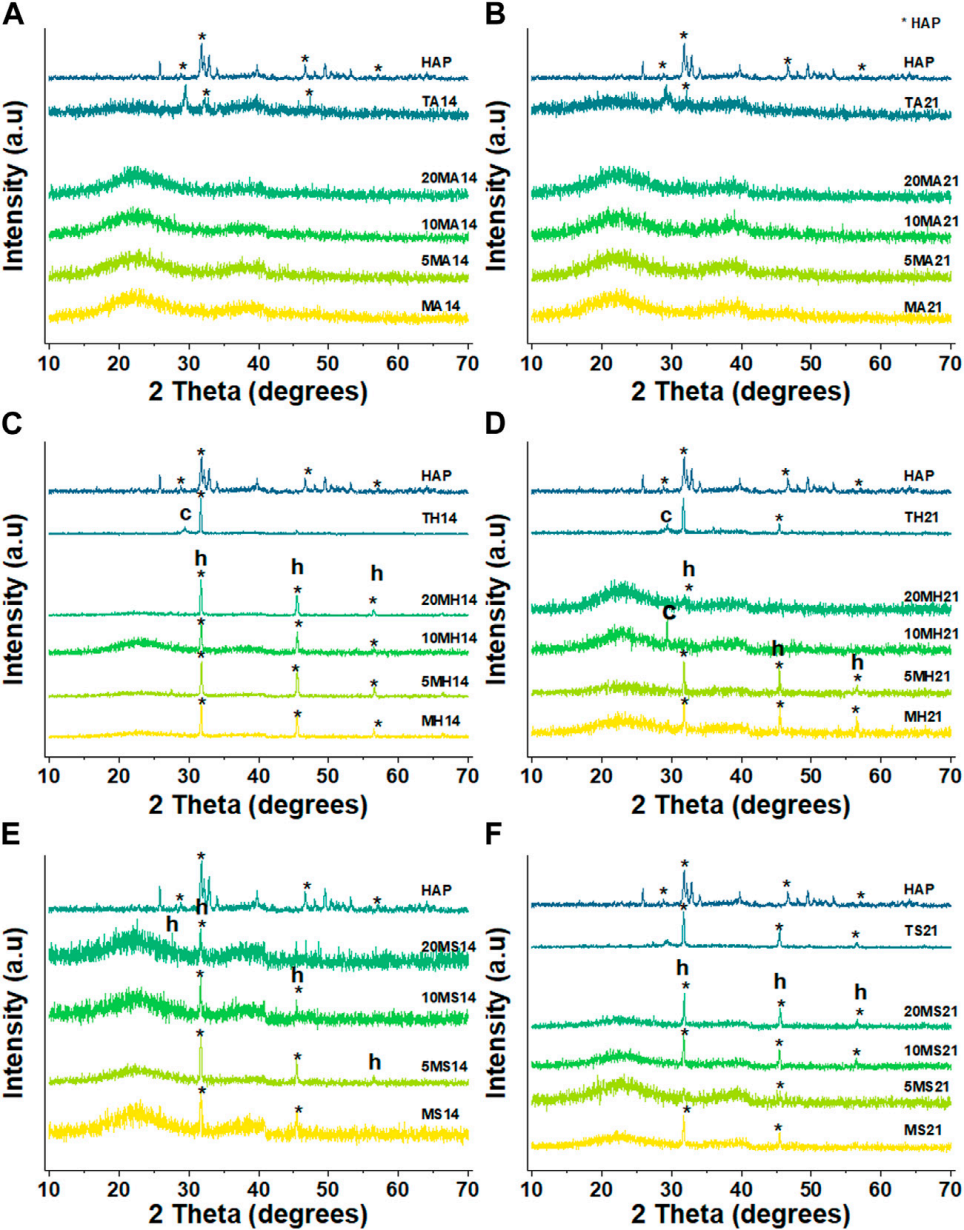
XRD patterns of the particles under investigation following 14 days and 21 dayays immersion in AS **(A and B)**, HBSS **(C and D)** and SBF **(E and F)** where * refers to the HAP, h represents halite, and c refers to calcite.

By contrasting the XRD patterns of the different particles in different immersion fluids at day 3, 14 and 21, the rate at which formation of apatite occurs can be determined. Figure 7 clearly reveals the difference in the behaviour of the particles following immersion for 3 days. In the case of AS, there appears to be no change in diffraction patterns among the MBGNs particles relative to the unimmersed particles (Figure 3). However, peaks associated with apatite begin to emerge at about 2θ 32° in HBSS and become even sharper and pronounced in SBF. At the same time, the original broad halo that typifies amorphous glass disappears at day three in SBF for all the MBGNs. In the subsequent data for 14 days and 21 days, shown in Figure 8 below, the intensity of the apatite signal increases in HBSS and SBF, but remains relatively unchanged for AS. Other than the apatite peak at 2θ 32°, there are two more peaks at around 45° and 56°. In contrast, samples immersed in AS fail to show much evidence of apatite formation. Unfortunately, some of the data is also too noisy and may be hiding some of the peaks, especially in the case of TCS. Although for all particles the most obvious peak is around the 2θ 32° diffraction angle, there are other equally important peaks that should be considered. For example, there are potential Ca(OH)_2_, CSH, DCS and TCS peaks which overlap in TCS making it harder to isolate the HAP phase. However, by considering these patterns alongside the delicate, sheet-like morphology interspersed with small apatite globule features obtained by SEM, this conclusion can be made. There is a tendency for undoped MBGNs to show more intense apatite peaks compared to 20LiMBGNs at the later time points. This is especially obvious in HBSS and SBF as seen in Figure 8 below.

**FIGURE 9:**
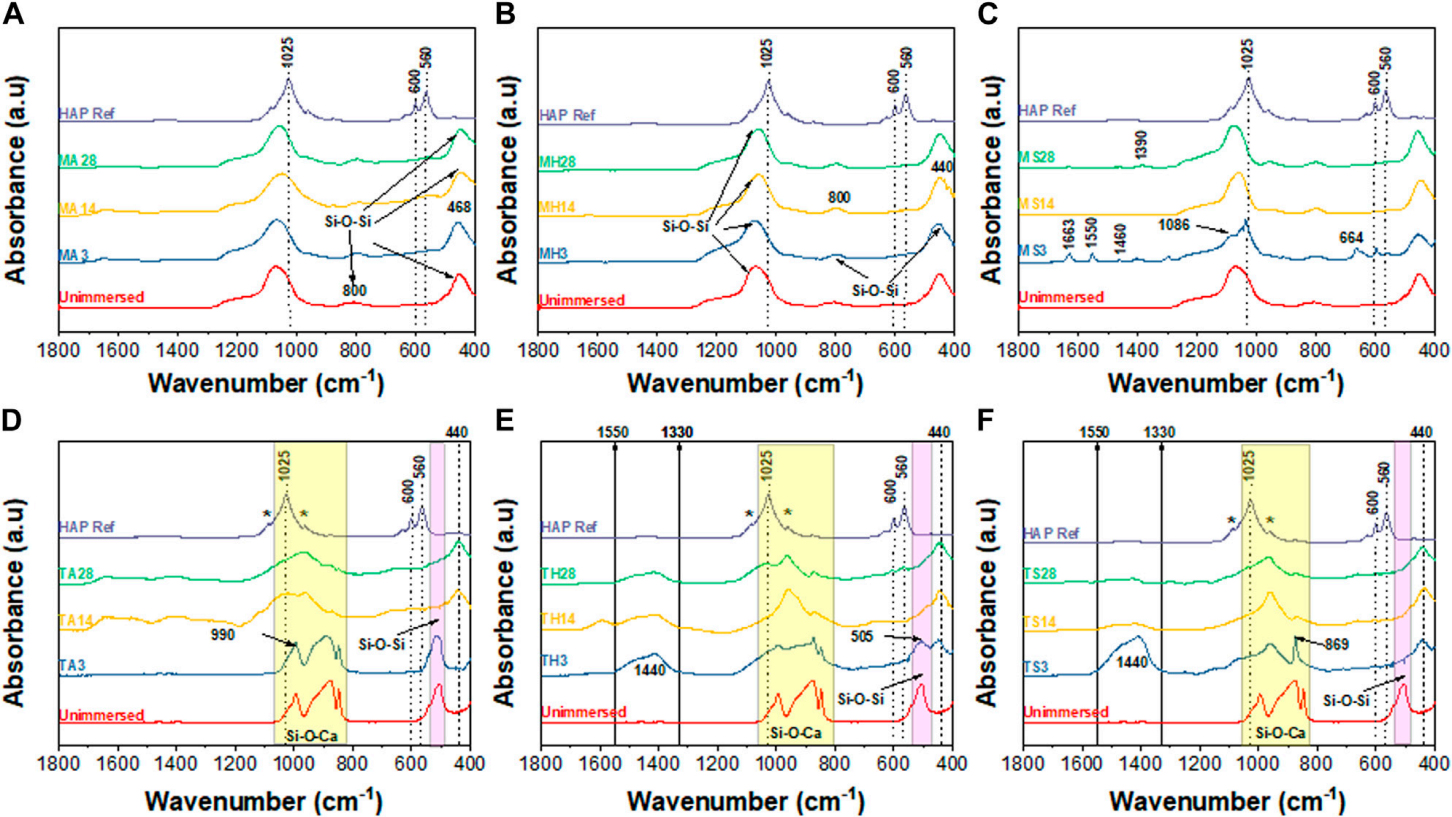
Combined FTIR spectra after immersion in the three media for 3 days, 14 days and 28 days. MBGNs in AS **(A)**, HBSS **(B)** and SBF **(C)** and TCS in AS **(D)**, HBSS **(E)** and SBF **(F)**.

**FIGURE 10:**
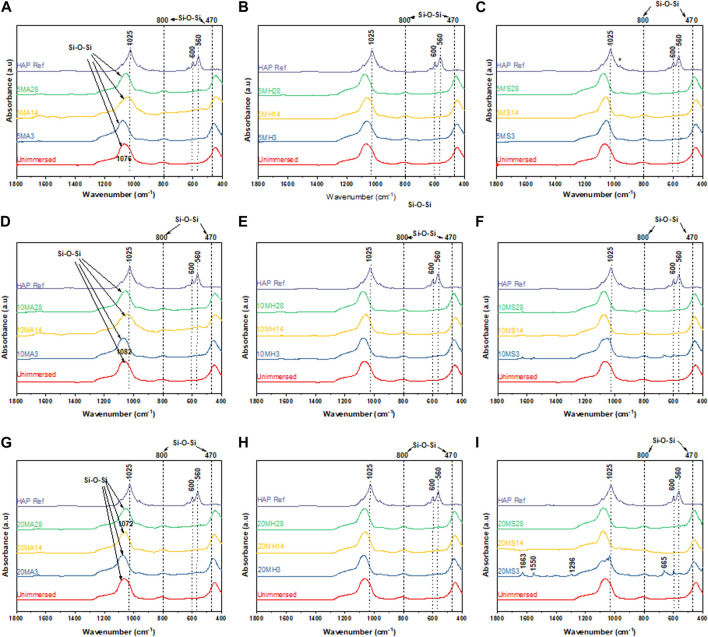
FTIR spectra for the doped glasses after immersion in the three media for 3 days, 14 days and 28 d 5LiMBGNs in AS **(A)**, HBSS **(B)** and SBF **(C)**; 10LiMBGNs in AS **(D)**, HBSS **(E)** and SBF **(F)**; and 20LiMBGN sin AS **(G)**, HBSS **(H)** and SBF **(I)**.

Figure 9 and Figure 10 summarize the infrared spectroscopy data in the 400–1800 cm^−1^ range following immersion for three, 14 and 28 days in the various media. For the TCS particles in Figure 9, the distinction between unimmersed and 3 days immersion in AS is not prominent. However, at day 14 and day 21 in AS, and at all the time points in HBSS and SBF, peak evolution occurs, highlighting the formation of various bonds. P-O bonds that can be attributed to hydroxycarbonate apatite (HCA) are identifiable at around 560 cm^−1^ and 605 cm^−1^ (Filgueiras et al., 1993). These may also be characteristic of the presence of orthophosphates. TCS displays a disappearance of the peak at 505 cm^−1^ in SBF by day 3, however it is still present in HBSS and AS. In its place new peaks emerge. These new peaks at around 440 cm^−1^ and a broad peak spanning 1330–1550 cm^−1^ may be attributed to carbonated compounds (Simila et al., 2017). Concurrently, TCS particles that were immersed in HBSS and SBF specifically show a sharp band at around 1440 cm^−1^ on day 3, which can be attributed to surface carbonates. This signal gradually broadens and flattens out at day 14 and day 21 probably signaling the formation of carbonated apatite or calcium hydroxide. The same span has split peaks among MBGNs, and these are especially pronounced for MBGNs, 10LiMBGNs, 20LiMBGNs at day 3 but appear absent thereafter. Generally, FTIR provided the least distinctions between the mesoporous nanoparticles, except for MBGNs, 10LiMBGNs and 20LiMBGNs, whereby visible peaks are especially obvious in SBF, at day 3. Overall, the FTIR peaks associated with apatite can be seen at around wavenumber 606 cm^−1^, 1026 cm^−1^ and 1086 cm^−1^. The latter peaks are not marked on the plots as they are hardly detectable in most of the MBGNs spectra.

Generally, immersion of TCS results in bands corresponding to the apatite formation process to a greater extent than immersion of the MBGNs given that the detectable peaks for the former are much more intense, and are present in HBSS and SBF simultaneously. Therefore, we can report that both the particle type and immersion fluid played a role in determining the rate and adequacy of apatite crystallization over time.

### 4.4 Antimicrobial efficacy of MBGNs and TCS against *E.coli* and *S.aureus*

**FIGURE 11:**
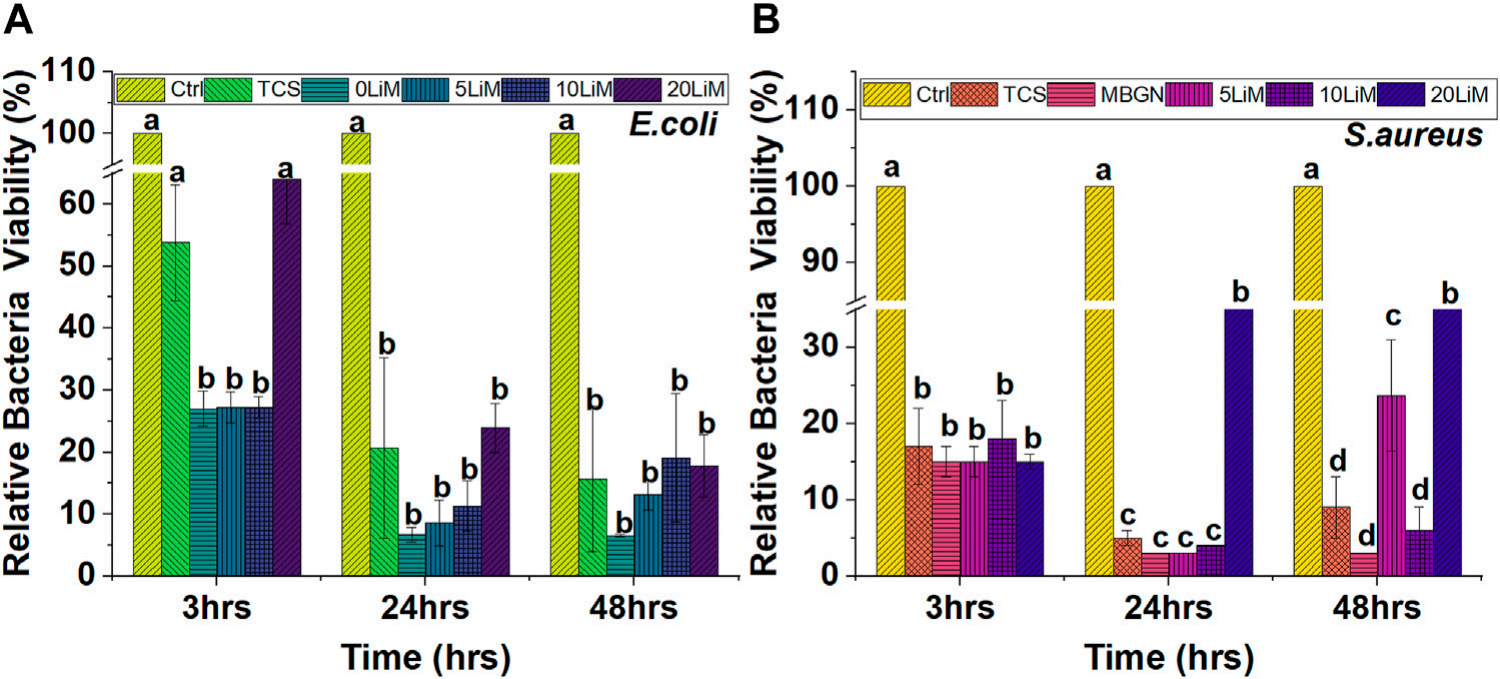
The effect of the particles on Gram negative—*(E) coli* **(A)** and Gram positive bacteria—*S. aureus* **(B)** after 3, 24 and 48 h. Dissimilar letters indicate means that are statistically different from each other. (*n* = 3; * = *p* > 0.05).

All particles showed significant inhibition of bacterial growth, but the effect was more pronounced for undoped MBGNs. The antibacterial effects are generally higher for *S. aureus* than *E. coli.* In Figure 11 below, the inhibitory effect against *S. aureus* is similar for all the five particles during the initial 3 h. At this timepoint, an average 85% reduction in bacteria viability is reported. However, at 24 h, the relative effect is pronounced for TCS, MBGNs, 5% and 10% doped MBGNs only, while it appears to decrease, and remains so for 20LiMBGNs even after 48 h.

With respect to *E. coli* in Figure 11, TCS and 20LiMBGNs had the least effect relative to the other particles especially during the 3 h and 24 h evaluations. At 3 hours, the *E. coli* viability corresponds to 54% and 64% for TCS and 20LiMBGNs, respectively, and proved to be statistically similar to the control, while being statistically significant different in relation to the remaining mesoporous nanoparticles. At 24 h however, the differences within the experimental groups were not statistically significant different from each other, but show significantly less bacterial growth compared to the control. At the same time, *S. aureus* growth was significantly reduced for all the particles at all time points, when compared to the control. On average, the overall antibacterial effect is prominent at 24 h, regardless of bacteria type.

### 4.5 Biocompatibility using MG-63 cells

**FIGURE 12:**
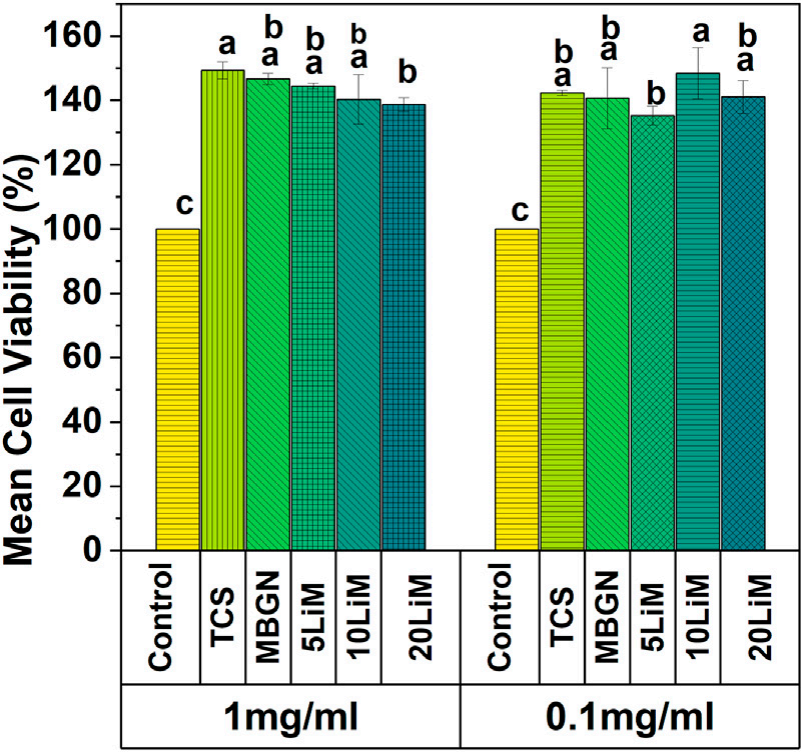
Relative cell viability of MG63 cells with two concentrations of sol-gel synthesized TCS, and MBGNs doped with various concentrations of Li at 48 h. Means that do not share a letter are significantly different from each other (*p* < 0.05).

Through the indirect cell viability assay, it was possible to investigate the impact of the particles on MG63 cell behavior. In Figure 12, we observe an enhanced viability of the cells at both concentrations (1 mg/mL and 0.1 mg/mL). Regardless of the particle type, the cell viability is significantly higher, compared to the control. The average viability of TCS is 149% at 1 mg/mL which is the highest among all the tested groups at this concentration. Additionally, there is a significant difference between TCS and 20LiMBGNs at 1 mg/mL concentration, as well as between 5LiMBGNs and 10LiMBGNs at 0.1 mg/mL.

### 4.6 Haematoxylin and eosin (H&E) staining

**FIGURE 13:**
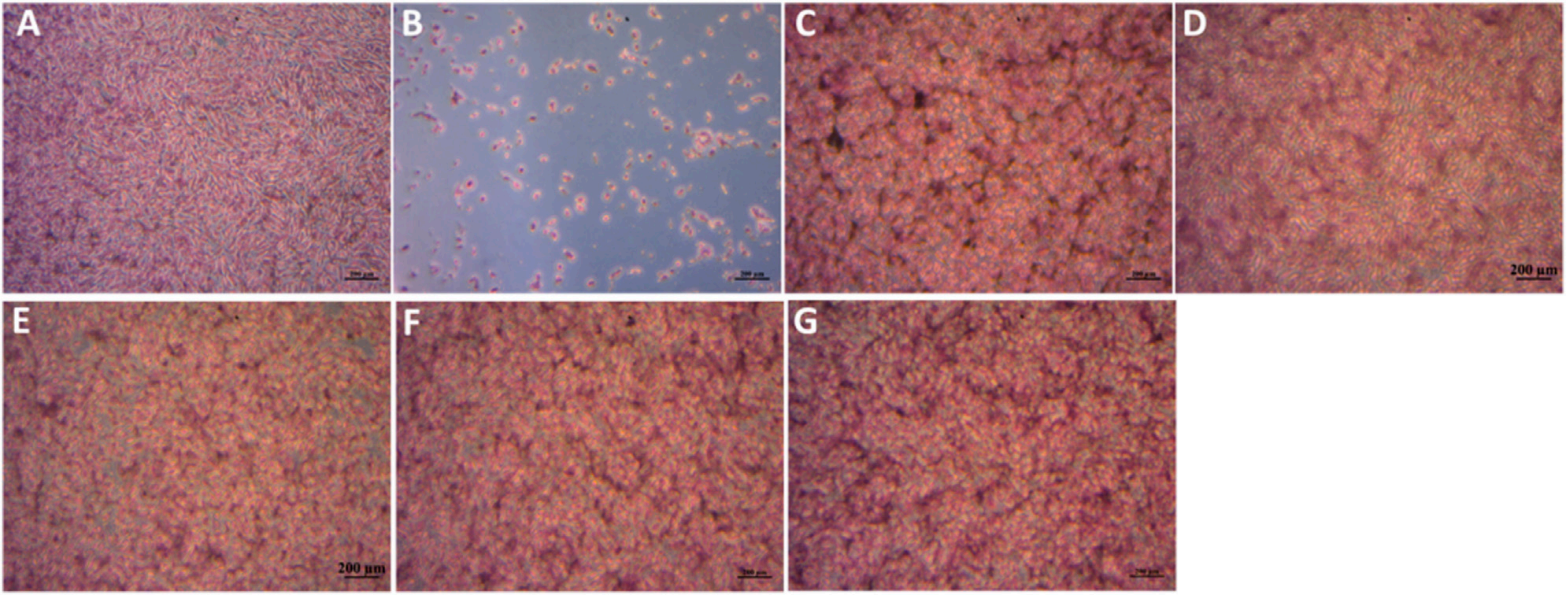
MG63 cells stained by H& E after incubation with sample extracts at a concentration of 1 mg/mL for 48 h. **(A)**- Pos Ctrl, **(B)**- Neg Ctrl, **(C)** TCS, **(D)**- MBGNs, **(E)**− 5LiMBGNs, **(F)**- 10LiMBGNs, **(G)** 20LiMBGNs.

Following H& E staining of the MG63 cells, optical microscopy images of the cells which had earlier been co-cultured with the particle extracts for 48 h were taken as shown in Figure 13. The results are congruent with the cell viability data as evidenced by well spread cells on the bottom of the well plates, which appear to be firmly attached. The overall quantity and quality of cells treated with glass eluates matches that of the positive control (A) while being distinctly different from the negative control (B). Notably, the TCS group appears rounded, tightly packed with some regions of overlap. This behaviour is consistent with their excessive multiplication within the limited well plate surface area.

## 5 Discussion

Biomaterials that are responsive to the aggressive oral environment and can drive biomimetic dentistry represent a vibrant area of research (Forsback et al., 2004; Gandolfi et al., 2011). Development of such materials commences with synthesis and testing of select precursors which can eventually be incorporated in various dental materials (Skallevold et al., 2019; Makvandi et al., 2020; Simila and Boccaccini, 2022). The aim of this research was to synthesize TCS, MBGNs and Li-doped MBGNs by the sol-gel method and perform comparative investigations that could rank their performance where certain properties for target dental application are of interest. We especially sought to characterize the synthesis outcomes of the particles under study, followed by determination of the chemical behaviour of the produced particles when exposed to AS, HBSS and SBF over a certain time span. Further, we checked the potential for antimicrobial effects against two important microorganisms. Finally, we investigated how osteoblast cells respond to eluates from these particles as a baseline comparison of biocompatibility.

TCS particles in this study were irregularly shaped and ranged in size from 120 to 240 nm. This is a departure from the 2.21 μm–7.53 μm sizes established by Balbinot et al. (2020) and a range of 1.73 μm–69.54 μm reported elsewhere (de Cesare et al., 2021). This can be explained by the fact that in the mentioned studies, post synthesis particle size reduction was achieved by manual grinding, whereas in the current study, equipment assisted milling of the particles was carried out. Morejón-Alonso et al. (2011) also measured a particle size under 10 μm, although the exact size is not specified. An alternative explanation of these contradicting results is the type of strategy employed in measuring the particle size. Dynamic light scattering particle size analysis was the common method used, probably due to ease and convenience. However, this method has been shown to artificially increase particle size especially if the particles are prone to agglomeration (Yeap et al., 2018). Our results however match the 200-nm–sized particles in a study by Huang et al. (2017). Elsewhere a particle size of 4–7 nm is reported (Radwan et al., 2019). For the studies reporting particle sizes in the micron range, there is a possibility that this was the size of the agglomerates, as opposed to the individual particles. In our experience, irregularly shaped agglomerates were imaged by SEM at lower magnifications, but what appeared to be separate nanoparticles could be observed at higher magnifications. Moreover, initial attempts to use laser light for particle size determination proved unreliable with the device generated error report suggesting a high probability of agglomeration. The challenge in completely resolving and separating these particles can be explained by high electrostatic interaction that overcomes any amount of shearing forces applied to separate the nanoparticles.

All MBGNs were relatively monodispersed uniform spheres which is typical of the modified Stöber method of sol-gel synthesis (Zheng et al., 2016; Zheng and Boccaccini, 2017). The presence of mesopores on the particle surfaces is consistent with the use of CTAB as a shape directing agent in microemulsion assisted method of sol-gel synthesis (Neščáková et al., 2019). Overall, our attempts to synthesize Li-doped MBGNs by sol-gel yielded particles whose morphology and microstructure did not appear to be adversely affected by this ion dopant as proven by absence of any crystallization peaks in Figure 3. This is in agreement with a similar study by Moghanian et al. (2017). The size range of these MBGNs falls within the expected nanometre range for MBGNs of 20–500 nm (Greasley et al., 2016). Although the mean sizes of the doped MBGNs were slightly smaller than undoped MBGNs, with the smallest size being linked to 5LiMBGNs, the overall size variation was not statistically significant within the mesoporous particles (Figure 2). There was no clear pattern to the size reduction hence the precise effect of Li ion precursors on MBGNs dimensions cannot be explained. From the ICP-OES data, we note that although Li incorporation is achieved, it appears to be much lower than the nominal concentrations expected. This is not unusual and is one of the challenges associated with producing ion doped MBGNs (Zheng et al., 2019). It is possible that the sol-gel stirring steps may not have allowed sufficient incorporation of this small and light ion into the overall structure of the glass. Alternatively, a lot of the same ion may have been lost during the final washing steps which has been the case in explaining the generally lower Ca ion incorporation in standard Ca-Si MBGNs (Greasley et al., 2016). Generally, although successful production of Li-doped MBGNs by sol-gel process is reported in literature (Moghanian et al., 2017), details regarding actual Li ion incorporation are not discussed.

Analysis of the XRD patterns and FTIR spectra of the MBGNs calcinated at 700°C reveals similarities (Figure 3). All the spectra of the unimmersed MBGNs have clear peaks at around 450 cm^-1^ and 1070 cm^-1^ which represent Si-O-Si rocking and Si-O-Si stretching vibrations, respectively (Aguiar et al., 2018; Kurtuldu et al., 2021). There is uniformity in the broad peak spanning the 780 cm^-1^ - 820 cm^-1^ wavelength range which is consistent with the Si-O-Si wagging vibration. The intensity of this peak can be used to interpret the presence and extent of non-bridging oxygen (NBO) bonds affected by doping (Nawaz et al., 2018). In this study however, the intensity of this peak remains unaffected and is consistent with low concentrations of dopant which are unlikely to alter the molecular structure of the MBGNs. This is typical of doped MBGNs hence it is challenging to confirm Li ion incorporation by FTIR. Additionally, the glass matrix itself tends to obscure any absorption bands of the dopant considering that the glasses have a Si-O-Si bending vibration at the 800 cm^-1^ region. XRD patterns indicate that the amorphous nature of the MBGNs was maintained regardless of the Li concentration introduced. Thus, on the basis of the FTIR spectra and XRD patterns collected, no significant changes to the structure of these MBGNs can be attributed to the dopant. The FTIR and XRD results of the as synthesized TCS particles are also consistent with previous studies. The FTIR peaks at around 505 cm^-1^, 880 cm^-1^ and three split peaks spanning the 820 cm^-1^—1060 cm^-1^ are typical of Si-O-Ca bonding (Balbinot et al., 2020).

Even though the MBGNs particle morphology was not prominently affected by ion doping, obvious distinctions between the TCS and MBGNs particles and even within the MBGNs subgroups begin to emerge when we consider the pH behaviour (Figure 4) and apatite formation (Figure 7). Overall, there were some changes to the pH and bioactivity behavior based on the particle type and the presence of Li ion. The association between pH changes of immersion media and apatite formation has been reported in multiple studies (Cerruti et al., 2005; Björkvik et al., 2016; Arango-Ospina et al., 2019; Khalid et al., 2021). This validates the approach of the present study in which pH and bioactivity in AS, HBSS and SBF are compared and contrasted in order to provide more robust data regarding the properties of these particles in relation to this important chemo-biological parameter. We especially wanted to distinguish how the fewer ions in AS and its proteinaceous nature are unlike HBSS and SBF whose heavily ionic nature that readily drives apatite precipitation should not be entirely relied upon for evaluating bioactivity of particles targeted for oral applications (Bohner and Lemaitre, 2009; Pan et al., 2010; Baino and Yamaguchi, 2020).

Generally, pH elevation was less pronounced in AS and highest in HBSS. Parallel bioactivity data also shows that detection of apatite was least within the AS experimental groups. This is not surprising since greater apatite formation tends to correlate with high pH (De Aza et al., 2005). It is reasonable to speculate that low amounts of P and high protein content in saliva, impair the ability of bioactive particles to form apatite. Mucin has especially been cited as a deterrent to apatite formation through complex formation at certain sites of the mucin protein that deprives the system of sufficient Ca to react with P (Boskey, 1996; Lagúnez-Otero et al., 2002). This compromise in bioactivity following immersion in AS is more obvious for MBGNs. More specifically, undoped MBGNs recorded generally higher pH than their highly doped 20LiMBGNs counterpart in all the fluids. This may be as a result of the effect of Li on release of Ca hence impeding the latter’s buffering ability. Additionally, 20LiMBGNs obviously had a compromised Ca release profile due to lower bioavailability of Ca in the synthesized particles (Table 2). The above can explain what appears to be the decreasing intensity of peaks associated with apatite, with increasing Li content seen in the XRD patterns of Figure 8. Alternatively, it could be that Li substitution also decreases the amount of Si present by precipitating Si in a SiO_2_ rich layer which impedes further glass dissolution (Moghanian et al., 2017). Thus, longer immersion times are not necessarily translating to more apatite formation among the doped glasses after 3 days. The observed attenuation in bioactivity is also reflected in a different study on 10% Li doped 45S5 BAG (Miguez-Pacheco et al., 2016). Although the study was performed on melt-quench glasses and scaffolds, the rationalization behind the reduced apatite forming ability can still apply to our sol-gel derived particles. An even more appropriate study on Li-doped sol-gel derived MBGNs also resulted in reduced bioactivity with increasing Li substitution (Khorami et al., 2011). It is also possible that the same rational of high Zn substitution resulting in unstable apatite, may be at play here such that the Li ion which has a smaller ionic radius compared to Ca causes lower presence of crystalline apatite at increasing time points (Mutlu et al., 2022). However it is more plausible to attribute decreased degradation and bioactivity to the more stable glass structure arising from the Li ion substitution (Brückner et al., 2016), which has been observed to result in denser glass and glass ceramic structure (Maçon et al., 2017). Slowing or actual retardation of bioactivity associated with different ion substitutions has been reported in literature (Watts et al., 2010; Hoppe et al., 2011; Moghanian et al., 2017; Neščáková et al., 2019).

We additionally suggest that the generally low pH of MBGNs in AS compared to the surge recorded for TCS comes about from the susceptibility of glass to degradation at low pH. This in turn can explain why there was almost no apatite formation among the MBGNs when immersed in AS since any high degradation rate hinders precipitation of apatite from Ca and P (Cerruti et al., 2005). The overall high pH detected with the TCS particle is possibly arising from the greater Ca release from this particle which favoured the obvious apatite detected with these particles even in AS, the low baseline pH notwithstanding. It is worth noting that the higher pH values measured for TCS relative to the MBGNs are generally less obvious following immersion in SBF.

Taking an in-depth look at the bioactivity results of the TCS particles, FTIR data show distinct time dependent differences between day three, 14 and 28, summarized in Figure 9. Some of the peaks consistent with apatite are highlighted with an * to coincide with 1080 cm^-1^ and 960 cm^-1^. The more prominent apatite peaks at around 1025 cm^-1^, 600 cm^-1^ and 560 cm^-1^ have been also marked on the HAP reference spectrum included in all the graphs. Additional peaks can be seen specifically as an increase in intensity, with increasing immersion time at around 870 cm^-1^ and in the 1440–1460 cm^-1^ range. These peaks are assigned to carbonated apatite and have already been observed among TCS based dental cements (Simila et al., 2017). These data is further supported by the accompanying XRD results in Figure 7 and Figure 8 and the SEM results in Figure 6. Although we would have expected to see an apatite peak at 26 2θ, this was not the case possibly because TCS based dental cements that display this specific peak also tend to contain calcium carbonate which contributes to formation of carbonated hydroxyapatite (HAP). However, the peak line highlighted at around 46 2θ is also indicative of carbonate (Hench et al., 2014). Within the framework of this experiment, this is likely due to interaction with the CO_3_ present in the immersion fluids. Regarding the same TCS results observed, it needs to be emphasized that although the highlighted XRD peaks in the 32 2θ area may also represent TCS and DCS crystals, the presence of apatite like features in the SEM images and FTIR bands associated with PO_-4_, gives us relative confidence in assigning apatite to these peaks.

When it comes to the bioactivity of the mesoporous particles, suspected apatite formation can be detected in HBSS and SBF. However, in light of the negligible confirmation of PO_4_ bonds by FTIR analysis, the mere presence of the intense peak at 32 2θ in the XRD patterns could not be attributed to formation of only HAP. Indeed, given the nature of these immersion tests, compounds such as calcite and vaterite could be expected to supersaturate in moderate amounts (Drouet, 2013). Therefore, repeat XRD and Rietveld refinement on two exemplary samples was performed and this confirmed the presence of not just calcite, but halite peaks as well, superimposed over the apatite peak positions. (Supplementary Figure S1). Porous glasses synthesized by the sol-gel method often precipitate carbonate on their surface as alternatives to or in addition to apatite (Maçon et al., 2015). Unfortunately, this has only been critically reviewed in a handful of recent research papers (Mozafari et al., 2019; Baino and Yamaguchi, 2020). It is suspected that the halite may have precipitated from the immersion media itself considering that sodium chloride makes up the greatest fraction of SBF and HBSS (Table 1). Trace amounts of MgS0_4_.7H_2_O were also detected. Apatite was not clearly visible in the glass immersed in HBSS, and was therefore not included in the Rietveld analysis of this sample. In spite of that, minor fractions of low crystalline apatite are surmised to be present.

On the other hand, changes associated with bioactivity are generally lacking in AS, even though this differs from other related research. This behaviour can be the result of the BAG chemistry and pH of the AS used. For example, significant apatite formation by a SiO_2_-CaO-CaF_2_-Na_2_O glass in AS has been reported elsewhere as early as 3 days (Khalid et al., 2021). Admittedly, the artificial saliva used was high in orthophosphates, which is known to contain 0.68 g/L of KH_2_PO_4_ and 0.856 g/L of Na_2_HPO4·12H_2_O 0.856 (Tamburrino et al., 2020). In contrast, our composition lacked the later and only contained 0.35 g/L of K_2_HPO_4_.3H_2_0. Aina et al. (2011) also tested various glasses in AS and reported pH changes of up to 3 units and apatite detection as early as 24 h. We suspect that this is likely due to the experiments involving a mucin free AS and the glass composition having Na and F which were absent in the present research (Aina et al., 2011). In the case of De Aza et al. (2005), use of stimulated saliva collected from healthy participants also resulted in irrefutable evidence of bioactivity in a diopside ceramic which lends evidence to the fact that AS cannot be assumed to be unsuitable for checking bioactivity of particles targeting the oral environment. It must be mentioned that in the aforementioned study, the pH of the saliva was eight and rose to a high of 9.8 within 1 h and remained at this level for a month. Similar apatite forming ability associated with AS is also reported in a study of sol-gel derived bioactive glass approximating tooth discs (Curtis et al., 2010). Although referencing melt-quench glasses, the fact that apatite could form in AS of pH four was surprising but is probably a factor of the Na and F presence in the said glass that favored pH increment and subsequent fluorapatite formation (Khalid et al., 2021). The contradictory results from the different studies we cite strengthen the rationale for the importance of both broad and specific evaluations of different bioactive particles and immersion fluids in order to obtain comprehensive data on different bioactive particles. The interplay of particle composition and specific saliva type in influencing overall bioactivity is appreciated better through such approaches. Although all sol-gel derived particles showed a degree of bioactivity, the difference in appearance and rate of apatite crystals results from the different compositions and microstructures which in turn influence the dissolution rate, pH changes and nucleation of new crystals (Aina et al., 2011; Arango-Ospina et al., 2019; Lepry et al., 2019). Through this experimental approach we show that reacting TCS and MBGNs bioactive particles in relevant test media that have different pH and ion concentration, is a better strategy for understanding the course of dissolution and precipitation that is pertinent for site specific application of such nanoparticles.

Our series of particles all demonstrated a degree of growth inhibition of two bacteria - each representing a Gram-positive or a Gram-negative bacteria strain (Figure 11). Although our basic screening of TCS vs. MBGNs was performed on *E. coli* and *S. aureus*, the outcomes are informative for predicting how other bacteria that play a more prominent role in dental infections such as *Streptococcus mutans* (*S. mutans*), *Lactobacilli casei* (*L.casei*) and *Enterococcus faecalis* (*E. faecalis*) could be affected by these particles. The two tested microorganisms were selected because they are typical indicators for monitoring antibiotic resistance (Boss et al., 2016).

Although all the particles show an antibacterial effect compared to the control, without particle eluate, our results fail to unveil any superior inhibitory effect of MBGNs doped with Li. If anything, the highest percentage of Li, correlates with higher bacterial viability which is pronounced for *S. aureus*, even after 24 h and is also observed at 3 h, with *E. coli* (Figure 11). The inhibitory effect of TCS was lower than that of MBGNs, although a level of inhibition was still measured relative to the control at all time points and for both bacterium types investigated. Generally, the relative bacterial viability is lowest at 3 h, drops further after 24 hours, before rising again slightly after 48 h.

Generally, BAG nanoparticles are known to lead to bacteria toxicity by elevating pH, causing Ca/P precipitation on the bacteria surfaces and by increasing osmotic pressure (Shrestha and Kishen, 2016; Ray and Dasgupta, 2020). In the present study, we observed that the doped MBGNs were not significantly more antibacterial than MBGNs. 5LiMBGNs had the lowest density of bacteria among the doped glasses for all time points and bacteria, except at 48 h for *S. aureus* where both 5% and 20% Li-doped MBGNs had statistically significant numbers of viable bacteria compared to the undoped and 10% LiMBGNs. It is possible that among the MBGNs, the pH played a greater role in inhibiting bacterial growth than the extent of ion doping. Although the pH of the bacterial medium was not measured, the pH measurements in AS, HBSS and SBF were typified by declining alkalization of the immersion fluids from MBGNs to 5, 10 and 20LiMBGNs. Therefore, if a similar pattern was occurring in bacterial broth, then higher antibacterial efficiency is expected at low Li concentrations or in the absence of this dopant. Historically, the potential of Li containing compounds to arrest infection was comprehensively reviewed for the first time by Lieb some 15 years ago (Lieb, 2007). It was proposed that this occurred through inhibition of prostaglandins that occur during infection. Li ion in particular has been shown to inhibit *E. faecalis* to a greater extent compared to undoped BG (Kavitha et al., 2014). Although the study referenced differed from ours in the testing methodology (agar diffusion method), type of bacteria and the inclusion of phosphate ions in the Li doped glasses, the results can be adapted to our own findings. In another case, multi drug resistant *S. aureus* was inhibited by BG doped with 5% lithium to a greater extent than 10% lithium (Moghanian et al., 2017). This goes to show that an exponential increase in antibacterial efficiency is not necessarily guaranteed by higher ion substitution. Similar trends were established in our experiments whereby at most time points, the highest bacterial viability among the MBGNs was linked to the 20LiMBGN. Since Li is thought to impart antibacterial properties, failure to prove antibacterial superiority of LiMBGNs over the undoped counterpart warrants further investigation. When considered alongside the pH studies and the proposal that Li stabilizes the glass structure (Moghanian et al., 2017), then a plausible explanation for the lower antibacterial effect observed could be the reduced degradation rate of the more stable glass. Notably, other studies have also shown that an antibacterial effect is not always proven for doped MBGNs, even when doped with ions such as Zn and Nb which are known to inhibit bacteria (Uskoković et al., 2021).

The evidence on the capability of TCS particle to inhibit bacteria is divided (Janini et al., 2021). On one hand, some studies indicate that these particles impact bacteria by hydroxyl ion release and alkalinisation which in turn damage the membranes of bacteria cells (Bueno et al., 2016). On the other hand though, no such effect can be proven (Jardine et al., 2019; Pelepenko et al., 2020). Part of the controversy has been attributed to the test methods applied whereby use of the agar disc diffusion method is often clouded in misinterpretation of the halo caused by diffusion of soluble ions (Farrugia et al., 2017). In addition, the pH argument fails to adequately explain why the TCS, which had the highest pH during immersion studies, has the least inhibitory effect in our study. This adds to the lack of clarity regarding the mechanism of action of TCS particles on bacteria and calls for extended investigations of this phenomenon.

Elsewhere, a recent study showed that Li doped glasses have a greater antimicrobial effect than undoped glass or TCS (Ali et al., 2022). We have to point out that the study results could have differed from our own on the basis of the test methodology, since actual cements containing TCS or lithium surface pre-reacted glass (S-PRG) particles were investigated in that study (Ali et al., 2022), unlike in our case where individual particles were evaluated. Elsewhere, excellent antimicrobial activity against a variety of oral bacteria was also reported for sol-gel synthesized 45S5 BAG that was doped with Li (Palza Cordero et al., 2021). The fact that the said study only considered BAG doped with 5% Li ion, may indicate that antimicrobial benefits of this ion are sufficient at concentrations as low as 5%. The overall greater antibacterial effect of MBGNs relative to TCS could be emanating from their smaller particle size hence enhanced surface area to mass ratio which improves the overall reactivity of these particles (Curtis and Wilkinson, 2001).

In terms of cytocompatibility, we utilized the indirect method as outlined in the ISO-10993–5 norm and observed enhanced cell viability for all the particles after 48 h compared to the control (Figure 12). The enhancement occurred at both 1 mg/mL and 0.1 mg/mL concentrations. The images of the H&E staining (Figure 13) also confirm that all particles tested were not toxic to the cells, thus being in harmony with the WST results. Although TCS particles are statistically significant more favourable for cell growth than 20LiMBGNs at the higher concentration, no significant differences were found relative to the other MBGNs at this concentration. At the lower concentration, however, there seems to be a significant difference in the cell viability of 5LiMBGNs and 10LiMBGNs.

In general TCS particles have been shown to be highly biocompatible (de Oliveira et al., 2018). In both *in vitro* and clinical studies, these particles have consistently resulted in favourable outcomes, the high pH not withstanding (Primus et al., 2019). For example, an evaluation of TCS cements containing increasing amounts of calcium tungstate particles concluded that TCS without additive resulted in the highest cell viability both before and after hydration setting reactions (Balbinot et al., 2020). Related studies also confirmed the good biocompatibility of this composition (Lee et al., 2018; Abdalla et al., 2020). In general, the cytocompatibility observed with these particles is likely arising from the role of Ca which is known to activate calcium sensors and channels allowing Ca influx into the cell and subsequent cell proliferation (Borowiec et al., 2014; Athanasiadou et al., 2018; Chen et al., 2019). The comparative viability of the synthesized particles in our study matches recent findings by Ali et al. (2022), who also reported on the relative biocompatibility of a TCS based dental cement, and lithium surface pre-reacted glass (S-PRG) fillers (Ali et al., 2022). The favourable effect of Li on cells has also been replicated in recent work that was specific to sol-gel derived Li substituted 58S BAG (Moghanian et al., 2017). MBGNs have routinely been considered to favour cell growth and proliferation (Amudha et al., 2020) even though the suitability, comparability and interpretation of biological studies by different authors is debatable (Salètes et al., 2021). At the same time, although a clinical study on sol-gel BAG for pulp capping attributes the negative outcomes of the said BAG to the biodegradability and high pH of the glass (Elhamouly et al., 2021), our results demonstrate that a high pH should not necessarily result in unfavorable sequalae. We observed that despite TCS particles being responsible for the highest increase in pH, TCS supported cell viability as well. It should be kept in mind that the *in vitro* environment of our study in which MG63 cells were employed, differs from an *in vivo* environment where materials are in contact with odontoblast cells. That notwithstanding, our current results provide a rational basis to conduct subsequent studies that employ pulp stem cells and in doing so, these studies contribute to the direct comparison of the two compounds as recommended by the authors of the clinical trial (Elhamouly et al., 2021).

Our own study agrees with the general consensus of the cell enhancing properties of MBGNs while also proving that Li doping does not negatively affect cell viability. Not surprisingly, Li doping was also proved to be non-cytotoxic in studies by Khorami et al. (2011), Han et al. (2012) and da Silva et al. (2017). To strengthen the understanding of the comparative profiles of these particles, it would be prudent to consider specific cells that participate in dentine pulp regeneration, higher concentrations of the eluate and a wider variety of time points at which the cell viability is tested. Evaluation of the ion release profile and correlation to the pH of the cell culture medium and cell viability are important tasks for future consideration.

## 6 Conclusion

Within the limits of this study, TCS and Li doped MBGNs were successfully synthesized by sol-gel method and systematically compared. Whereas the MBGN particle size and morphology fell within a range of 120–200 nm without post synthesis processing, TCS required significant milling procedures to obtain particles that were in a similar size range as the MBGNs. Additionally, TCS particles were associated with higher alkalinizing effects following immersion in various fluids and greater bioactivity, while MBGNs exhibited better antimicrobial properties, which makes synergizing these effects in dental biomaterial compositions, attractive. The fact that both particle groups are biocompatible favour such synergism. However, the decreased apatite forming ability of MBGNs in AS observed in our study needs to be investigated further. Perhaps, it may be desirable to conduct similar experiments while using silico-phosphate and phosphate containing MBGNs and compare the behaviour in AS to our current phosphate free MBGNs. This is more so for the target application of the synthesized particles that call for greater interaction with saliva, as opposed to dentinal tissue fluid which can be easily mimicked by SBF and HBSS. It seems that introduction of Li into 70Si30Ca MBGNs alters glass microstructure resulting in the unique pH and apatite formation behaviour discussed in the present paper. Additionally, there may be an alternative explanation for the higher antimicrobial effect of MBGNs, besides pH, which should be scrutinized further. Overall, varying immersion media presents realistic data on how bioactive particles react in oral fluids, which is relevant when considering them as additives in specific dental materials. In future, in depth studies of experimental dental cements and composites that incorporate these particle types in various proportions are planned to further clarify the observations presented in this study.

## Data Availability

The raw data supporting the conclusion of this article will be made available by the authors, without undue reservation.
