# Supplementary material for: Sol-gel synthesis of lithium doped mesoporous bioactive glass nanoparticles and tricalcium silicate for restorative dentistry: Comparative investigation of physico-chemical structure, antibacterial susceptibility and biocompatibility
Source: Front Bioeng Biotechnol. 2023 Apr 3;11:1065597. doi: 10.3389/fbioe.2023.1065597 (PMC10106781; doi:10.3389/fbioe.2023.1065597)
Supplement: Supplementary file 1 [file Table1.DOCX]

Supplementary Material

**Table 1: Comparative synthesis and processing of MBGNs and TCS particles**

| NPs | Composition & Precursors | Preparation steps & time | Gelation | Drying | Calcination | Additional processing |
| --- | --- | --- | --- | --- | --- | --- |
| MBGNs **(**Li**)** | DI, CTAB, EA, NH_4_OH,TEOS, Ca(NO_3_)_2_.4H_2_O Li.NO_3_.4H_2_O | 5-6 stages of reagent addition and stirring, followed by 4 rounds of rinsing and centrifugation:**7 hrs** | During stirring @ room temp | 60°C @ 24hours | 700°C for 3 hours | None |
| TCS | DI, H_2_NO_3_, Ca(NO_3_)_2_.4H_2_O | 3 stages of reagent addition and stirring: **2hrs 40min** | Overnight @ 60°C | 120°C @ 24hours | 700°C for 24 hours | Milling and sieving |
