# Supplementary material for: Sol-gel synthesis of lithium doped mesoporous bioactive glass nanoparticles and tricalcium silicate for restorative dentistry: Comparative investigation of physico-chemical structure, antibacterial susceptibility and biocompatibility
Source: Front Bioeng Biotechnol. 2023 Apr 3;11:1065597. doi: 10.3389/fbioe.2023.1065597 (PMC10106781; doi:10.3389/fbioe.2023.1065597)
Supplement: Supplementary file 2 [file Image1.PDF]

Supplementary Material

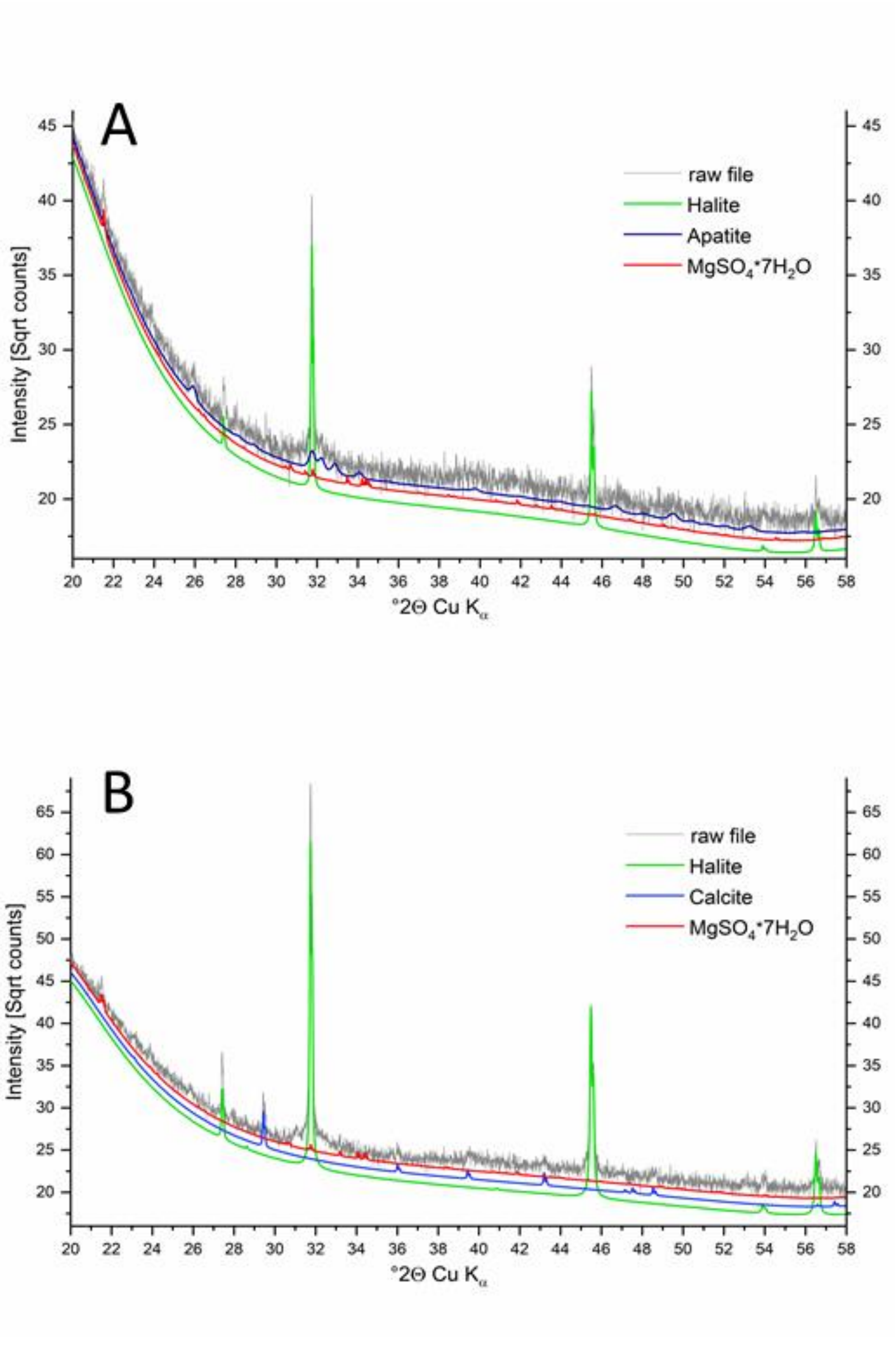

***Supplementary Figure 1**: XRD pattern of lithium doped MBGN, immersed in SBF for 21 days (20MS21) (A); and MBGN, immersed in HBSS for 14 days (MH14) (B) after Rietveld refinement.*
